# Renal cell carcinoma in young *FH* mutation carriers: case series and review of the literature

**DOI:** 10.1007/s10689-019-00155-3

**Published:** 2019-12-02

**Authors:** J. A. Hol, M. C. J. Jongmans, A. S. Littooij, R. R. de Krijger, R. P. Kuiper, J. J. T. van Harssel, A. Mensenkamp, M. Simons, G. A. M. Tytgat, M. M. van den Heuvel-Eibrink, M. van Grotel

**Affiliations:** 1 Princess Máxima Center for Pediatric Oncology, Heidelberglaan 25, 3584 CS Utrecht, The Netherlands; 2 Department of Genetics, University Medical Center Utrecht/Wilhelmina Children’s Hospital, Utrecht, The Netherlands; 3 Department of Radiology, University Medical Center Utrecht/Wilhelmina Children’s Hospital, Utrecht, The Netherlands; 4 Department of Pathology, University Medical Center Utrecht, Utrecht, The Netherlands; 5 Department of Genetics, Radboud University Medical Center Nijmegen, Nijmegen, The Netherlands; 6 Department of Pathology, Radboud University Medical Center Nijmegen, Nijmegen, The Netherlands

**Keywords:** Hereditary leiomyomatosis, Fumarate hydratase, Renal cell carcinoma, Children, Adolescents, FH mutation, HLRCC

## Abstract

Hereditary Leiomyomatosis and Renal Cell Cancer (HLRCC) is an autosomal dominant syndrome caused by heterozygous pathogenic germline variants in the fumarate hydratase (*FH*) gene. It is characterized by cutaneous and uterine leiomyomas and an increased risk of developing renal cell carcinoma (RCC), which is usually adult-onset. HLRCC-related RCC tends to be aggressive and can metastasize even when the primary tumor is small. Data on children and adolescents are scarce. Herein, we report two patients from unrelated Dutch families, with HLRCC-related RCC at the ages of 15 and 18 years, and a third patient with an *FH* mutation and complex renal cysts at the age of 13. Both RCC’s were localized and successfully resected, and careful MRI surveillance was initiated to monitor the renal cysts. One of the patients with RCC subsequently developed an ovarian Leydig cell tumor. A review of the literature identified 10 previously reported cases of HLRCC-related RCC in patients aged younger than 20 years, five of them presenting with metastatic disease. These data emphasize the importance of recognizing HLRCC in young patients to enable early detection of RCC, albeit rare. They support the recommendations from the 2014 consensus guideline, in which genetic testing for *FH* mutations, and renal MRI surveillance, is advised for HLRCC family members from the age of 8–10 years onwards.

**Electronic supplementary material:**

The online version of this article (10.1007/s10689-019-00155-3) contains supplementary material, which is available to authorized users.

## Introduction

Hereditary leiomyomatosis and renal cell cancer (HLRCC) is an autosomal dominant syndrome caused by heterozygous germline variants in the fumarate hydratase (*FH*) gene, associated with an increased risk of developing renal cell carcinoma (RCC).

The first report describing a family with HLRCC was published in Finland in 2001, and over 300 affected families from various countries have been described since [1–4]. Other clinical manifestations of HLRCC include multiple cutaneous leiomyomas in 73–100% of *FH* mutations carriers and uterine leiomyomas in ± 75% of female carriers [4–6]. Additionally, germline mutations in the *FH* gene have been identified in a small percentage of patients with paragangliomas and pheochromocytomas [7, 8].

The *FH* gene, located on chromosome region 1q42.1, is a tumor suppressor gene that encodes the enzyme fumarate hydratase (fumarase), which plays a role in both the tricarboxylic acid (TCA) cycle in mitochondria, as well as the response to DNA double strand breaks in the nucleus [3, 9]. Somatic inactivation of the second allele can be demonstrated in most, but not all, HLRCC-related tumors [2, 10–12]. Biallelic germline mutations are rare and cause a syndrome known as fumarase deficiency, characterized by early onset, severe encephalopathy [5]. In patients with fumarase deficiency, mutations are usually missense or in-frame duplications that do not necessarily result in complete loss of enzyme activity [13]. More than 200 distinct variants spread over the entire coding region of the *FH* gene have been published in the Leiden Open (source) Variation Database system (LOVD) [13] and so far, a clear correlation between the type or location of the *FH* mutation and cancer risk has not been observed [5].

The absolute risk of developing RCC is estimated to be 10–15%, with a median age of onset of 40–41 years [4, 14]. RCC can be the first manifestation of HLRCC. Histologically, loss of staining for FH and positive staining for 2-succino-cysteine (2SC), which accumulates in the setting of FH deficiency, can support the diagnosis of HLRCC-related RCC [14, 15].

In adults, HLRCC-related RCC is known to be aggressive and can metastasize even when the primary tumor is small. Data on children and adolescents are scarce. We herein report three young patients from unrelated Dutch families, aged 15, 18 and 13 years respectively, as well as the results of a systematic literature review on HLRCC-related RCC in patients younger than 20 years. This review contributes to existing recommendations for genetic testing, tumor surveillance and resection in children and adolescents.

## Methods

Patients were evaluated at the Princess Máxima Center for Pediatric Oncology (case 1 and 3) and Radboud University Medical Center Nijmegen (case 2). Genetic, radiological and histopathological studies were reviewed. All patients as well as the parents in case 1 and 3, gave informed consent for inclusion of their clinical data in this manuscript.

For the literature review, databases of PubMed and Embase were searched for HLRCC-related renal tumors occurring in patients < 20 years (Supplementary Table 1). After removing duplicates, the search yielded 1221 articles (Supplementary Fig. 1). Any report (manuscript or conference abstract), written in English, Dutch, German, French or Spanish, describing a HLRCC-related renal tumor in a patient younger than 20 years of age, was eligible for inclusion. After title/abstract screening, a total of 86 reports were eligible for full text screening, during which 77 articles were excluded based on full text not being available, only including patients ≥ 20 years old, only reviewing or describing previously reported patients, or lack of germline genetic testing to confirm the diagnosis of HLRCC.

## Case presentation

### Case 1

**Fig. 1:**
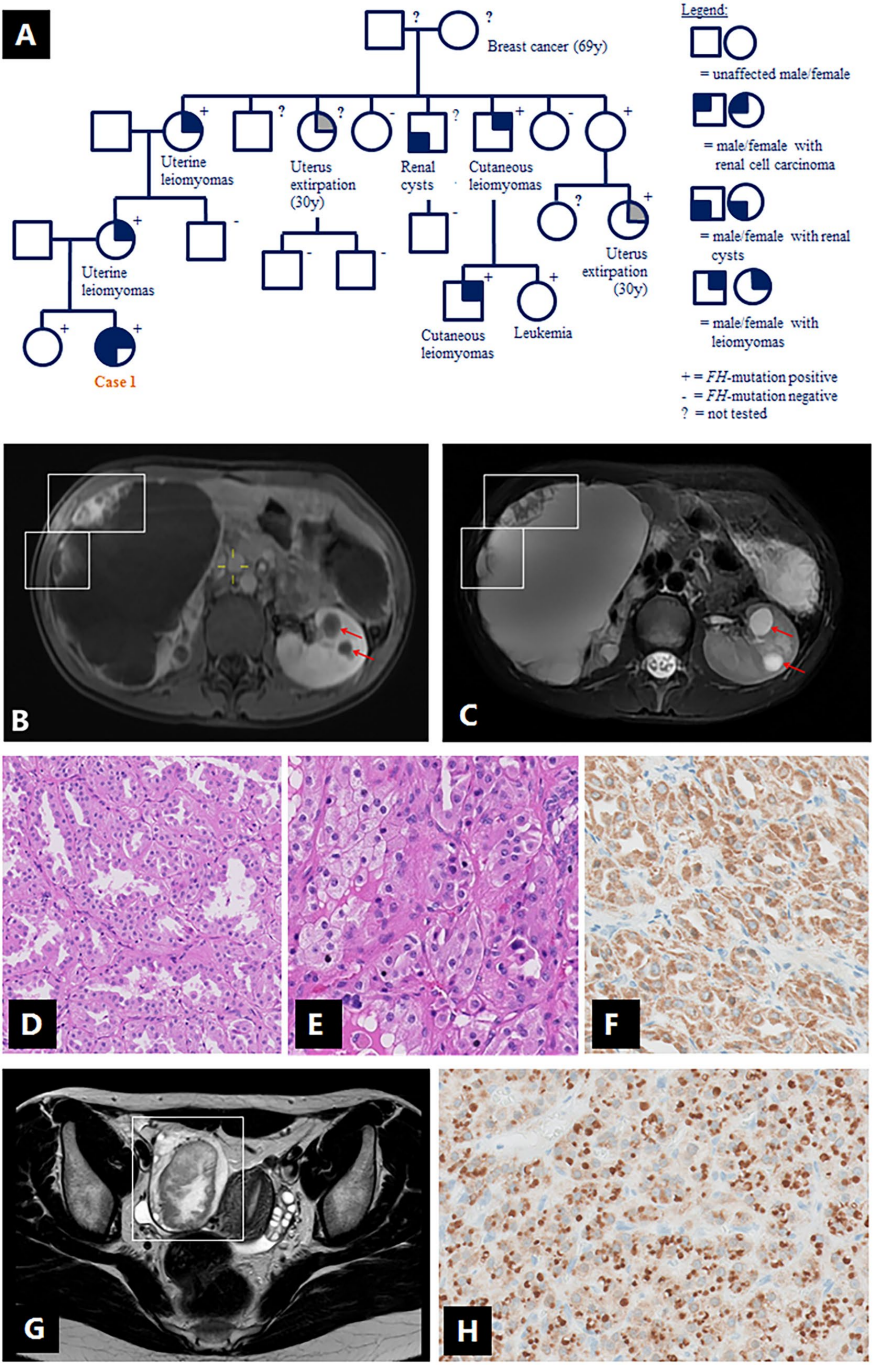
Case 1 (female, 15 years, renal cell carcinoma and Leydig cell tumor): **a** family pedigree; **b–c** contrast-enhanced T1W-MRI (**b**) and abdominal T2W-MRI (**c**) showing large right-sided kidney mass, which is mostly cystic with peripheral solid nodules (boxes). In the left kidney, multiple cystic lesions (arrows) are observed without solid components; **d–f** histology of the renal tumor: vital epithelial tumor with a predominantly tubular, partially papillary growth pattern (**d**) of strongly eosinophilic cells with mild to moderate nuclear atypia (**e**), and diffuse 2SC staining (**f**). **g** T2W-MRI of the pelvic region showing a right-sided ovarian lesion (box) with both solid and cystic components. **h** Ovarian Leydig cell tumor showing diffuse 2SC staining

A 15-year old female presented with a large right-sided abdominal mass. Her family history included uterine and cutaneous leiomyomas and a confirmed *FH* mutation in mother’s family (Fig. 1a). Physical examination revealed small, cutaneous lesions of the lower legs, suggestive for leiomyomas. On MRI using a customized HLRCC-protocol (Table 1), the mass was mostly cystic with peripheral solid nodules (Fig. 1b, c). The nodules showed strong enhancement after contrast administration and restricted diffusion on diffusion-weighted imaging (DWI). In the left kidney, multiple cystic lesions were observed without solid components. Brain MRI and total body FDG-positron emission tomography (FDG-PET) did not reveal signs of metastatic spread. Right-sided nephrectomy revealed an RCC with a maximum diameter of 20 cm (T2N0M0, four lymph nodes sampled), with tumor cells lining the cysts. There was no spread beyond the kidney and resection margins were free of tumor. Solid areas consisted of vital epithelial tumor with a predominantly tubular, partially papillary growth pattern of strongly eosinophilic cells with mild to moderate nuclear atypia (Fig. 1d, e) and diffuse 2SC staining (Fig. 1f). Prominent nucleoli were seen only in rare areas with papillary architecture, without perinucleolar halos. Germline genetic testing by MLPA confirmed the presence of the familial heterozygous deletion of the *FH* gene (c.(?_1)_(*1_?)del) in the patient and her 18-year old healthy sister, a deletion which has been previously reported in other patients with HLRCC [3, 6, 16–18]. The left kidney is monitored with MRI’s at 3 and 6 months after diagnosis, then every 6 months for 3 years, and yearly thereafter. Whereas the kidney appeared unchanged, the patient developed an ovarian lesion (Fig. 1g) after a follow-up of 30 months, at the age of 18, which was successfully resected and histologically characterized as a Leydig cell tumor; a well-demarcated lesion with uniform cells showing large, round nuclei, prominent nucleoli and lack of necrosis, nuclear atypia or mitotic figures. The tumor showed diffuse 2SC staining (Fig. 1h). Whole exome sequencing (Illumina NovaSeq platform) was performed on the Leydig cell tumor, but a second hit in the *FH* gene was not identified.

**Table 1:** Scan parameters and surveillance schedule for renal MRI surveillance in patients with HLRCC

Parameter	T2w-fat suppression	3D-T2	DWI	T1 pre/post
Pulse sequence	2-D MultiVane with spectral fat saturation	3-D turbo spin-echo with variable flip angle	2-D single-shot spin-echo with spectral fat saturation	2-D ultrafast spoiled gradient echo with fat suppression
Repetition time (ms)	2450	449	1611	3,89
Echo time (ms)	100	90	79	1,82
Flip angle (degree)	90	–	90	10
Slice orientation	axial	axial	axial	axial
Slice thickness (mm)	3	0.9–1.15	5	4
Slice gap (mm)	3	0	0	2
Echotrain length	30	85	1	1
Field of view (mm^2^)	400	400	450	380
B-values (s/mm^2^)	–	–	0, 150, 1000	–

### Case 2

**Fig. 2:**
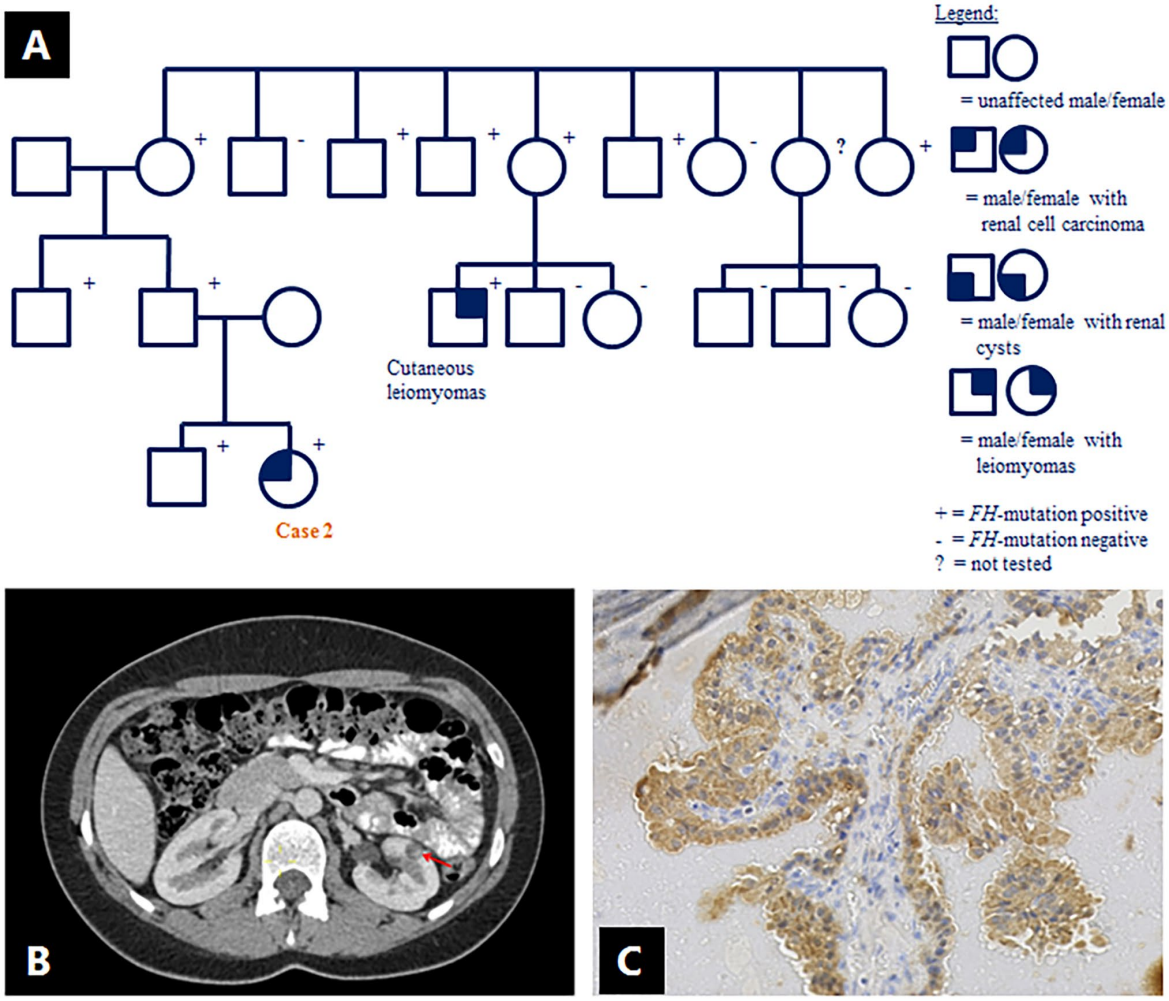
Case 2 (female, 18 years, renal cell carcinoma): **a** family pedigree; data are missing on the presence of leiomyomas in *FH* mutation carriers; **b** abdominal CT after contrast administration, showing a 9 mm cystic lesion in the left kidney, with an area of increased density (arrow) suspect for nodular enhancement. **c** Tumor cells showing diffuse 2SC staining

An 18-year old female, carrier of an *FH* mutation (c.1330delA; p.Arg444 fs; NM_000143.3), was referred for a suspect lesion in the left kidney, observed on renal MRI surveillance. The mutation was derived from her asymptomatic father and had been previously identified in a distant adult cousin with cutaneous leiomyomas (Fig. 2a). This mutation has not been previously reported. Subsequent CT-imaging with contrast administration showed a 9 mm cystic lesion, with an area of increased density suspect for nodular enhancement (Fig. 2b). A chest X-ray did not reveal signs of lung metastases. A partial nephrectomy was performed; the resected cyst showed focal papillary proliferations with a lining of atypical epithelial cells with some prominent nucleoli. The nucleoli were not significantly enlarged, strongly eosinophilic or surrounded by halos. No necrosis or strong mitotic activity were present. 2SC immunohistochemical staining was positive (Fig. 2c), and the lesion was characterized as an early stage of HLRCC-related RCC. A second hit analysis was not performed. The patient is doing well after a follow-up of 45 months.

### Case 3

**Fig. 3:**
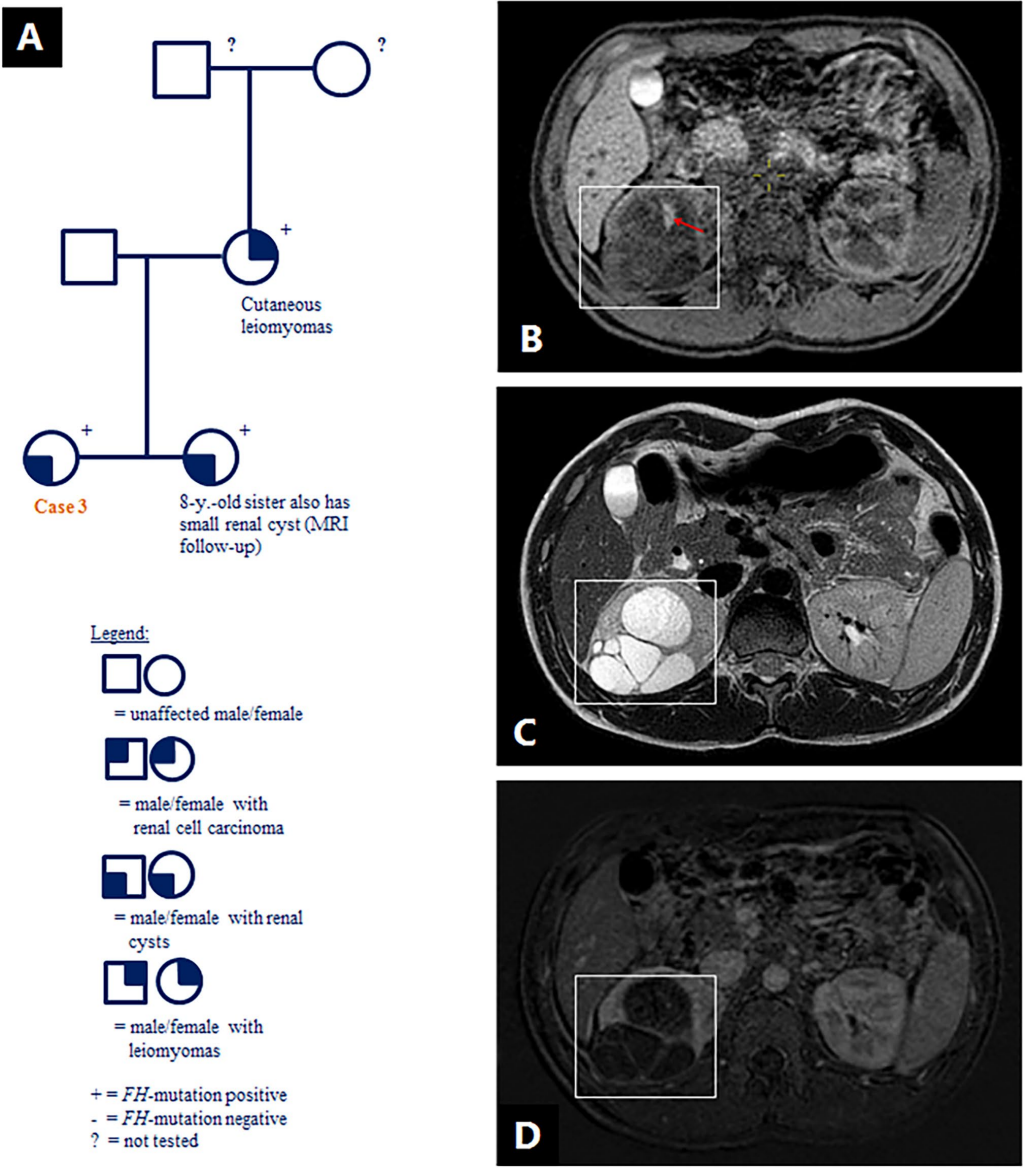
Case 3 (female, 13 years, complex renal cysts): **a** family pedigree; **b–d** abdominal T1 MRI (**b**) showing multilocular cysts (box) in the right kidney with area suspect for hemorrhagic content (arrow). Abdominal T2W MRI (**c**) and subtraction MRI (**d**) showing no nodular enhancement

A 13-year old female and her 8-year old sister were referred for ultrasound screening because of a recently confirmed *FH* mutation (c.1210G>T; p.Glu404*; NM_000143.3). The *FH* mutation was initially detected in the girls’ mother who had cutaneous leiomyomas (Fig. 3a), and this specific mutation was previously published in a case series [19]. In the 13-year old girl, the ultrasound identified two lesions in the right kidney which required further assessment, and the suspicion of RCC was discussed with the family. Subsequent MRI demonstrated two complex cystic lesions with variable hemorrhagic content in the right kidney with a maximum diameter of 7.1 cm and 2.2 cm respectively (Fig. 3b–d). No nodular enhancement was detected. An international review of the MRI scans agreed with this interpretation. After 18 months follow-up, the cysts had grown in size but no solid components appeared, with MRI’s performed at 3, 6, 12 and 18 months after the initial referral.

## Literature review

The literature review revealed 10 additional patients with HLRCC-related RCC diagnosed between 10 and 18 years of age (Table 2) [12, 18, 20–25]. Additionally, a Wilms tumor was identified in a 2-year-old female patient who later developed cutaneous and uterine leiomyomas at the age of 25. She was confirmed to carry a germline c.1189G>A (p.Gly397Arg; NM_000143.3) mutation in the *FH* gene. Since no tissue from the Wilms tumor was available, FH expression could not be evaluated and the causal relationship remains uncertain [26]. This particular mutation has been described in other patients with HLRCC, including the 11-year old patient with HLRCC-related RCC in Table 2 [22].

**Table 2:** HLRCC-related renal cell carcinoma (RCC) before the age of 20 years (confirmed *FH* mutation)

#	References	*FH* mutation	Age	Sex	Presentation	Histology	Disease stage	Outcome (FU)
1	Alam et al. [17]^a^	n.a.	16	F	Symptomatic	Collecting duct tumour	Metastatic	Died (2 years)
2	Merino et al. [12]^a^	n.a.	17	F	*NA*	HLRCC-associated RCC	Localized	*NA*
3			18	F	*NA*		Metastatic	*NA*
4	Al Refae et al. [21]^a^	c.1293del (exon 8)	17	M	Symptomatic	Papillary type 2 RCC	Metastatic	Died (15 months)
5	Alrashdi et al. [22]^a, b^	c.1189G>A (exon 8)	11	M	Surveillance	Papillary type 2 RCC	Localized	NED (3 years)
6	Gardie et al. [18]^a, c^	c.1123del (exon 8)	17	M	*NA*	Papillary type 2 RCC	Metastatic	Died (2 years)
7	Van Spaendonck-Zwarts et al. [23]^a^	c.1002T>G (exon 7)	18	F	Symptomatic	Papillary type 2 RCC, focally showing prominent nucleoli surrounded by a clear halo	Metastatic	Died (8 months)
8	Nix et al. [24]^d^ (*meeting abstract)*	n.a.	10	*NA*	*NA*	RCC, not specified	*NA*	*NA*
9	Toubaji et al. [25] (*meeting abstract)*	n.a.	18	*NA*	*NA*	RCC, not specified	*NA*	*NA*
10	Bhola et al. [27**]**	c.1430-1437dup (exon 10)	15	F	Symptomatic	Tubulo-papillary carcinoma	*NA*	*NA*
11	This report	Whole gene deletion	15	F	Symptomatic	HLRCC-associated RCC	Localized	Second tumor (Leydig cell tumor), 2 years after initial diagnosis
12	This report	c.1210G>T (exon 8)	18	F	Surveillance	HLRCC-associated RCC	Localized	NED (4 years)

Mutations are described using NM_000143.3

*FU* follow-up time since diagnosis, *NED* no evidence of disease, *n.a.* not available

^a^Previously included in literature review by Van Spaendonck-Zwarts et al. [23]

^b^Follow-up data reported in Van Spaendonck-Zwarts et al. [23]

^c^Follow-up data reported in Van Spaendonck-Zwarts et al. [23] and Wong et al. [28]

^d^This 10-year old patient is also referred to in Menko et al. [14] (describes personal communication with Dr. Linehan)

In two of the described young patients with RCC, histology was not further specified [24, 25]. Among the other patients, two tumors were described as HLRCC-associated RCC with a variety of histological patterns [12], whereas four tumors were described as papillary type 2 RCC [18, 21–23], one as tubulopapillary RCC [27] and one as a collecting duct tumor [20]. Although most patients were symptomatic at presentation, an 11-year old male patient was diagnosed with localized RCC at his first surveillance visit [22]. Overall, five out of ten patients presented with metastatic disease [12, 18, 20, 21, 23], two had localized disease [12, 22], and disease stage was not reported for the remaining three. Follow-up data were available of five patients, of whom four died within 2 years after diagnosis [20, 21, 23, 28]. The one patient with localized disease and follow-up data, showed no evidence of disease after 3 years [22]. The exact mutation was specified in 5/10 cases, including single nucleotide deletions in exon 8 in two patients [21, 28], a missense mutation in exon 8 [22], a missense mutation in exon 7 [23], and a duplication in exon 10 [27].

## Discussion

Including the two new cases in this report, a total of 12 RCC’s have been reported to date in *FH* mutation carriers younger than 20 years of age. Its aggressive nature, as illustrated by our literature review, emphasizes the importance of early genetic testing and surveillance.

Recently, a large, national series of French patients with HLRCC was published, in which 34 (19%) out of 182 *FH* mutation carriers developed RCC [4]. In this study, *FH* mutation carriers were identified through the two national laboratories accredited for *FH* germline testing. It is remarkable that none of the tumors in the French cohort occurred before the age of 20 years, illustrating that this early manifestation of HLRCC is rare and our literature review is likely to be influenced by a publication bias. Nevertheless, it may well be that *FH* germline testing is not always performed when RCC occurs in young patients from families that are not yet diagnosed with HLRCC. Notably, these patients may not yet have developed the typical clinical features of HLRCC.

In these patients the young age at diagnosis of RCC and characteristics of the tumor can trigger awareness for an underlying syndrome. Tumor characteristics typically associated with HLRCC, include papillary type 2 RCC and prominent nucleoli surrounded by a clear halo [12]. Yet, a recent review on histopathological features of FH-deficient RCC, concluded that a complex architecture with multiple histological patterns was more characteristic than the presence of perinucleolar halos. Moreover, histological patterns other than papillary type 2 RCC predominated in 40% of cases [29]. Interestingly, focused genetic testing in 212 RCC’s registered in the Children’s Oncology Group, revealed three FH-deficient RCC’s that were initially classified as RCC-NOS, in patients aged 17–18 years [30]. Since germline genetic data are lacking for these patients, a diagnosis of HLRCC could not be confirmed. Yet, these studies demonstrate the value of FH/2SC-immunostaining and genetic testing in unclassified or morphologically complex RCC, in both children and adults. Currently, HLRCC-associated RCC is recognized as a separate category in the World Health Organization (WHO) classification of renal tumors [31].

Leydig cell tumors, as identified in case 1, have been previously described in three patients with HLRCC, including two males with testicular Leydig cell tumors and a female with bilateral steroid cell tumors and metastatic RCC [32, 33]. With this fourth patient, we provide further evidence for an association between HLRCC and Leydig cell tumors. The three previously reported patients each had a different missense mutation in *FH*, and in contrast to the Leydig cell tumor of case 1, loss of the wild-type *FH* allele was demonstrated in the two testicular tumors [31]. Immunostaining for FH or 2SC was not performed in the previously reported cases, while in our patient, both the RCC and the Leydig cell tumor showed 2SC positivity, as expected in FH-deficient tumors.

In the past, in The Netherlands, it was advised to start genetic testing of HLRCC family members at the age of 20 years, but this changed based on evidence of early-onset RCC in this syndrome, including an 18-year old Dutch female from a known HLRCC family who presented with metastatic RCC and died 8 months after diagnosis [23]. Five out of the 12 young patients in our case series and literature review, presented with symptoms, of whom three died of disease [20, 21, 23, 27]. International recommendations for genetic testing and renal tumor surveillance were published in a 2014 consensus guideline, following discussions during the Fifth Symposium on Birt–Hogg–Dubé syndrome and Second Symposium on HLRCC [14]. Based on the report of a 10-year old patient [14, 24], the guideline recommends to offer *FH* mutation testing to children of affected families from the age of 8–10 years onwards, and if positive to start annual renal MRI screening (Box 1) [14, 34].

MRI is preferred over abdominal ultrasound, because of the low sensitivity of ultrasound to detect small lesions [14]. MRI is also considered superior to CT-imaging because radiation is avoided, which is particularly relevant in this young age category, and because of a better soft tissue resolution to identify small nodules that may be present in cyst walls. A specific HLRCC MRI-protocol (Table 1) is recommended, using 1–3 mm slices through the kidneys. If solid lesions are detected, a surgical resection with wide surgical margins is warranted, independent of the size of the lesions, unlike other hereditary renal cancer syndromes where surgical intervention is only recommended for tumors that exceed 3 cm [14].

It is unclear to what extent renal cysts have the potential to undergo malignant transformation. In 2006, Lehtonen et al. observed a higher prevalence of renal cysts in *FH* mutation carriers compared to the general population, but they did not find RCC to be more frequent in *FH* mutation carriers with renal cysts, compared to those without renal cysts [35]. Since then, three reports have been published suggesting that renal cysts may represent a potential preneoplastic lesion of the HLRCC-related renal cell carcinoma, based on the presence of atypical cells [12, 36] or 2SC uptake [37] in the lining of resected cysts. Therefore, we recommend to intensify surveillance if renal cysts are detected in *FH* mutation carriers, using shorter intervals between scans (Box 1).

A potential downside of early surveillance is the anxiety it may cause to patients and their families, particularly when a suspicious lesion requires further assessment, as illustrated by case 3 in this report. The risk and benefit of surveillance needs to be balanced in individual cases, in fair communication with the parents (shared decision making), and requires referral to expert centers with multidisciplinary teams.

Overall, our findings suggest that the incidence of HLRCC-related RCC is low but not negligible in patients younger than 20 years of age, emphasizing the importance of early genetic testing and renal surveillance in HLRCC family members. These data support the recommendations from the 2014 consensus guideline on HLRCC, in which genetic testing for *FH* mutations, and renal MRI surveillance, is advised from the age of 8–10 years onwards.

**Table:** Box 1

Recommended schedule for renal surveillance in *FH* mutation carriers	
Yearly MRI scans from the age of 8–10 years onwards	
If renal cysts are detected, closer monitoring is indicated:	
1st year: at 3, 6 and 12 months after detection of cysts, if no solid nodules appear:	
2nd–4th year: every 6 months, if no solid nodules appear:	
5th year and onwards: yearly MRI scans	
If solid nodules are detected, perform brain MRI and total body FDG-positron emission tomography (FDG-PET) for staging (repeat 1 × after 3 months)	
References: Menko et al. [14] and personal communication with Dr. W.M. Linehan	

## Electronic supplementary material

Below is the link to the electronic supplementary material.

Supplementary material 1 (DOCX 14 kb)

Supplementary material 2 (DOCX 29 kb)
