# Supplementary material for: Renal cell carcinoma in young FH mutation carriers: case series and review of the literature
Source: Fam Cancer. 2019 Dec 2;19(1):55–63. doi: 10.1007/s10689-019-00155-3 (PMC7026215; doi:10.1007/s10689-019-00155-3)
Supplement: Supplementary file 1 — Supplementary material 1 (DOCX 14 kb) [file 10689_2019_155_MOESM1_ESM.docx]

**Supplementary Table 1.** Search terms used in the literature search

**Pubmed:**

| Carcinoma*[Title/Abstract] OR Cancer[Title/Abstract] OR Tumor[Title/Abstract] OR Tumour[Title/Abstract] | OR | hypernephroma*[Title/Abstract] OR RCC[Title/Abstract] OR Grawitz[Title/Abstract] OR "Carcinoma, Renal Cell"[Mesh] |
| --- | --- | --- |
| AND |  |  |
| Renal[Title/Abstract] OR Nephroid[Title/Abstract] OR Kidney[Title/Abstract] OR Papillary[Title/Abstract] OR “Collecting duct”[Title/Abstract] OR Hypernephroid[Title/Abstract] |  |  |
| AND | | |
| HLRCC[Title/Abstract] OR Leiomyom*[Title/Abstract] OR “fumarate hydratase”[Title/Abstract] OR "Fumarate Hydratase"[Mesh] OR “FH mutation”[Title/Abstract] OR Reed’s[Title/Abstract] OR "Leiomyomatosis"[Mesh] | | |

**Embase:**

| Carcinoma*:ab,ti OR Cancer:ab,ti OR Tumor:ab,ti OR Tumour:ab,ti | OR | hypernephroma*:ab,ti OR RCC:ab,ti OR Grawitz:ab,ti OR 'renal cell carcinoma'/exp |
| --- | --- | --- |
| AND |  |  |
| Renal:ab,ti OR Nephroid:ab,ti OR Kidney:ab,ti OR Papillary:ab,ti OR ‘Collecting duct’:ab,ti OR Hypernephroid:ab,ti |  |  |
| AND | | |
| HLRCC:ab,ti OR Leiomyom*:ab,ti OR 'fumarate hydratase':ab,ti OR 'fumarate hydratase'/exp OR 'FH mutation':ab,ti OR Reed*:ab,ti OR 'leiomyomatosis'/exp | | |
