# Supplementary material for: Renal cell carcinoma in young FH mutation carriers: case series and review of the literature
Source: Fam Cancer. 2019 Dec 2;19(1):55–63. doi: 10.1007/s10689-019-00155-3 (PMC7026215; doi:10.1007/s10689-019-00155-3)
Supplement: Supplementary file 2 — Supplementary material 2 (DOCX 29 kb) [file 10689_2019_155_MOESM2_ESM.docx]

**Supplementary Figure 1.** Flowchart of literature search (Updated April 4^th^, 2019), *N = number of reports.*

**Inclusion criteria:**

- Any report describing HLRCC-related RCC occurring in patients <20 years;
- English, Dutch, German, French or Spanish language

**Exclusion criteria:**

- Previously reported cases;
- Germline genetic testing not performed

**Pubmed**

N=758

**Embase**

N=1192

N = 1950

**Removal of duplicates:**

N = 1221

**Title/abstract screening**

N = 86

**Full text screening**

Exluded articles:

- Full text not available: N=7
- Adult cases (≥20 years) only: N=58
- Review: N=15
- Previously reported: N=2
- Germline testing not performed: N=5

N = 9
